# Evaluating Predictive Value of Preoperative Clinical and Imaging Findings on the Short-Term Outcome of Surgery in Patients Undergoing Lower Lumbar Discectomy

**DOI:** 10.7759/cureus.20772

**Published:** 2021-12-28

**Authors:** Alireza Tabibkhooei, Sayyed Ehsan Ziaei, Maziar Azar, Meysam Abolmaali

**Affiliations:** 1 Department of Neurosurgery, Rasoul Akram Hospital, Iran University of Medical Sciences, Tehran, IRN; 2 Department of Neurosurgery, Shefa Neuroscience Research Center, Khatam Alanbia Hospital, Tehran, IRN

**Keywords:** surgical outcome, imaging, lumbar disc, disc herniation, discectomy

## Abstract

Objective: The most common indications for spinal surgery are low back pain and associated disabilities caused by disc herniation. Given the high prevalence of low back pain, the critical nature of pain assessment in these patients, and knowledge about the influencing and predisposing factors, we sought to evaluate the clinical and radiologic findings associated with pain relief and postoperative recovery in patients who underwent bilateral lumbar discectomy.

Methods: From March 2016 to October 2020, a prospective cohort study was conducted. This study included adult patients with symptomatic disc herniation in the L4-L5 and L5-S1 segments who were candidates for bilateral discectomy. Before and after surgery, patients were evaluated for lumbar and radicular pain and the Oswestry Disability Index (ODI) score and at the four, 12, and 24-week follow-up. Meanwhile, a variety of demographic, clinical, and radiologic factors was collected and statistically analyzed.

Results: This study enrolled 30 patients. The average age of the patients was 41.2 years, with 22 males and eight females. Twelve of these patients had disc involvement in the L4-L5 region, while 18 had disc involvement in the L5-S1 region. Statistical analysis revealed that radicular pain and disability associated with low back pain significantly decreased following surgery (P=0.001). However, there was no significant reduction in back pain (P>0.05). Patients with a higher body mass index (BMI) and neurological claudication had a lower reduction in radicular pain (P<0.05). Moreover, a higher BMI and the presence of instability pain are associated with an increased likelihood of postoperative lumbar pain relief (P<0.01). Among the radiological variables, increased disc height was associated with a more rapid recovery from low back disability (P=0.003). Furthermore, a larger diameter of paraspinal muscles at the level of the herniated disc was associated with a more rapid improvement of lower back pain (P=0.021).

Conclusion: The use of discectomy in patients with lumbar disc herniation significantly alleviates postoperative and follow-up radicular pain. Age, BMI, neurological claudication, instability pain, and paraspinal muscle diameter played a role in postoperative pain relief. Increased disc height was associated with a more rapid decline in the ODI score. Future studies with larger sample sizes are recommended.

## Introduction

Intervertebral disc herniation refers to the displacement of disc material beyond its normal limits [1]. Protrusion (or broad-based herniation), extrusion (or narrow-based herniation), and sequestration (where the herniated fragment loses its connection to the rest of the disc) are the three main types of disc herniation [2]. If the disc herniates posterolaterally, the protruded disc may cause nerve damage and radicular pain in the limbs [3].

Lower back pain is a frequent complaint in outpatient orthopedic clinics and is the leading cause of activity limitation in people under 45. Around 80% of people will experience lower back pain at some point in their lives [4]. Lower back pain occurs at a 15-45 percent rate per year, and the primary cause is lumbar disc prolapse [4, 5]. Although disc herniation is more prevalent in the middle-aged population, the number of young patients with lower back pain and disc herniation has recently increased [6]. Despite its occurrence, disc herniation rarely results in severe neurologic complications or sciatica [4]. A recent review showed that the prevalence of sciatic symptoms is rather variable, with values ranging from 1.6% to 43% [7].

L4-L5 and L5-S1 discs are the most frequently affected [4], and although the pain is severe and causes significant morbidity [6], it is likely to resolve over time with appropriate conservative therapy.

In most cases of disc herniation, rest, analgesics, and physical therapy help alleviate pain. Other options for symptom relief include transforaminal or epidural corticosteroid injections. If the pain persists for more than four to six weeks or worsens despite treatment, the surgeon may consider surgical intervention if neurologic deficit and refractory pain develop [8]. Discectomy techniques have evolved over time, progressing from wide laminectomy to hemilaminectomy to inter-laminar fenestration. The traditional wide-open laminectomy has a higher morbidity rate than less invasive procedures. Micro-invasive surgery techniques (lumbar microdiscectomy) have primarily supplanted previous methods in some cases, resulting in less vertebral instability and a shorter recovery time [4].

Microdiscectomy has established itself as the gold standard in disc herniation surgery, even though some surgeons occasionally continue to perform a standard open discectomy. Kovacevic et al. found that both procedures had a favorable overall functional outcome. The advantage of microdiscectomy was a greater reduction in leg pain and a lower reoperation rate [9]. 

Incomplete pain relief following surgical treatment of lumbar disc disease is a daily challenge for neurosurgeons. The patients in this study were those for whom less invasive procedures were not be considered effective; therefore, we tried to predict the prognosis of the treatment in relieving pain preoperatively by clinical and imaging findings. The purpose of this study was to examine the characteristics of patients with lumbar disc herniation, their imaging findings, and their overall outcome following bilateral discectomy surgery over six months. Furthermore, we examined whether the MRI findings were associated with disease characteristics and overall surgical outcomes.

## Materials and methods

This prospective cohort study was conducted on patients admitted to Rasoul Akram Hospital's neurosurgery department with lumbar disc disease, scheduled for elective bilateral discectomy surgery between April 2016 and April 2020. All operations were performed using a single technique and by a single neurosurgeon. The Iran University of Medical Sciences ethics committee approved all phases of this study. In this study, we used purposive sampling from among non-probability sampling methods.

Our study included all patients who met the following criteria: (1) Subjects who are over the age of 18 years; (2) Herniation (extrusion) of the lumbar discs at the L4-L5 and L5-S1 levels (we selected these two levels due to higher incidence and also considered that surgical discectomy in upper levels, especially for bulky herniated discs, would make instrumentation inevitable in some cases); (3) Patients scheduled to undergo bilateral discectomy; and (4) Patients who were not suitable for less invasive procedures due to the severity of disc herniation or neurological deficits. Patients with a history of skeletal or spinal abnormalities, prior lumbar disc prolapse surgery, or an inability to comply with follow-up were excluded.

For each patient, we evaluated the demographics (age, sex, BMI, preoperative pain duration), as well as clinical (straight leg raise test, neurological claudication, instability pain), and radiological data (paraspinal muscles area, disc height, grade Modic change, Pfirrmann grading system, degeneration in adjacent discs, osteophyte in vertebral body and effusion in vertebral facet). To our knowledge, this is the first study to evaluate the paraspinal muscles area as a variable in patients who underwent lower lumbar discectomy. For the measurement of the paraspinal muscles area, we evaluated the lumbar MRI images in the transverse plane at the level of the involved disc, using MARCO PACS diVision software (Tahavolat Novin Yademan Company, Tehran, Iran).

Patients were evaluated using MRI imaging methods. Baseline MRI studies of the lumbar spine were performed using a single device operating at 1.5 T, using a standard protocol turbo spin echo (TSE) or fast spin echo (FSE) sequences with sagittal and axial T1 and T2 weighted images [10]. The preoperative MRI assorted patients were evaluated for disc height, Modic changes [11], effusion within the facet, osteophyte in the vertebral body, paraspinal muscle dimensions, adjacent disc degeneration, and the Pfirrmann grading system [12].

Visual analysis of all MRI findings was performed in consensus by an experienced radiologist unaware of the patients' clinical data. Additionally, a detailed profile of patients' clinical characteristics was compiled, including their admitted demographics and clinical characteristics. During surgery, a coronal hemilaminectomy was performed on the involved segment. The herniated disc material was removed following Flavectomy, and loose disk fragments were evacuated bilaterally from the disk space using gentle medial retraction of the affected nerve roots.

The Oswestry Disability Index (ODI) questionnaire was used to assess functional outcomes. The primary outcome measure is the ODI score at preoperative and various follow-up visits. The study participants completed this questionnaire before surgery and four, twelve, and twenty-four weeks afterward. Moreover, patients were asked to complete a questionnaire regarding their low back and radicular pain using a visual analog scale (VAS) score concurrently with the ODI questionnaire [13]. No findings were detected in favor of obvious lumbar spine instability on MRI images. However, we evaluated spinal instability pain based on clinical tests for instability. These subjective measures included a history of painful locking during spinal motion (return from forwarding bending, transitional and trivial activity), problems with unsupported sitting, or pain that intensifies during sustained postures [14].

Statistical analysis

For descriptive analysis, the mean and standard deviation were used to describe continuous variables, while the number and regarded frequency were used to analyze categorical variables. The changes in ODI score, radicular pain, and low back pain preoperatively and at various follow-ups were analyzed using repeated measure analysis of variance (ANOVA). Additionally, multivariate linear regression was used to determine the factors associated with improving the ODI score, radicular pain, and preoperative low back pain following surgery. Statistical significance was defined as a P-value less than 0.05, and all statistical analyses were conducted using IBM SPSS Statistics for Windows, Version 22.0 (released 2013, IBM Corp., Armonk, New York). We used G*Power software to determine the sample size. The sample size calculation was conducted based on a study conducted by Li et al. [15].

## Results

For the final analysis, data from 30 patients were retrieved. The mean age of the participants was 41.2±12.6 years. The study population consisted of 22 (73.3%) males and eight (26.7%) females. The mean BMI was 25.6±2.8 (range 22-31) and 16 patients (53.3%) were overweighted or obese. Lower back pain was reported to have lasted an average of 6.8 months (ranging between one to 48 months). Among the participants, 12 (40%) and 18 (60%) had a disc herniation at the L4-L5 and L5-S1 levels, respectively. Table 1 contains information about the patients' demographics.

**Table 1:** Demographics of patients n: number;  op: operation

Variables		
No. of patients		30
Gender (n, (%))		
Male		22 (73.3)
Female		8 (26.7)
Age (years, means±SD)		41.2±12.6
BMI (kg/m^2^, means±SD)		25.6±2.8
Pre-op pain duration (months, (range))		6.8 (1-48)
Herniation level (n, (%))		
L4-L5		12 (40)
L5-S1		18 (60)

Of the 30 patients, 18 (60%) had neurological claudication and seven (23.3%) complained of instability pain. MRI evaluation revealed that seven patients (23.3%) had no visible endplate signal changes, five patients (16.7%) had grade I Modic changes, 13 patients (43.3%) had grade II Modic changes, and five patients (16.7%) had grade III Modic changes. Four patients (13.3%) were grade III, 14 patients (46.7%) were grade IV, and 12 patients (40%) were grade V, according to the Pfirrmann grading system for lumbar disc degeneration.

**Figure 1:**
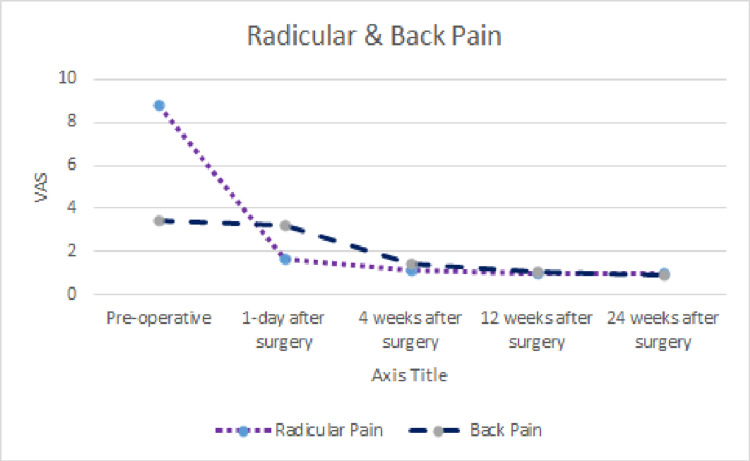
Trends in outcomes of the radicular and back pain evaluated in the study

**Figure 2:**
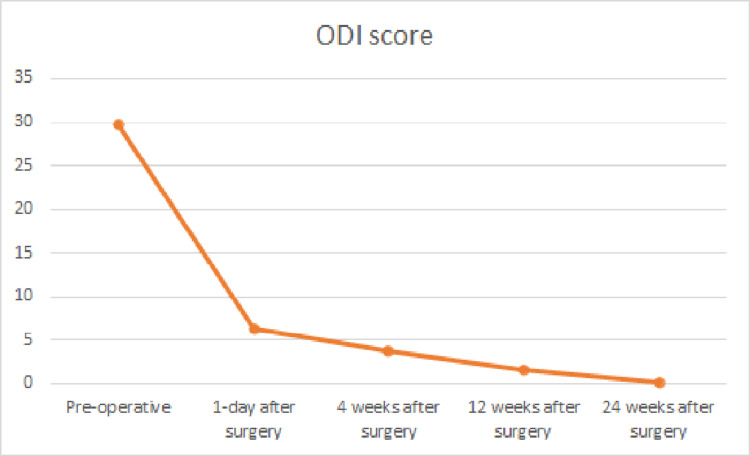
Trends in the outcomes of the Oswestry Disability Index (ODI) evaluated in the study

Table 2 summarizes the clinical and radiologic findings of the patients. Clinical symptoms such as radicular pain improved significantly postoperatively (Pain score: 8.8±1.15 vs. 1.63±0.67, respectively, P = 0.001) (Figure 1). However, postoperative back pain did not improve significantly (Pain score: 3.47±2.93 vs. 3.23±1.67, respectively, P =0.7) (Figure 1). Preoperative mean ODI was 29.8±5.2. The mean ODI at the first day, fourth week, 12th weeks, and 24th weeks postoperatively were 6.27±2.3, 3.73±2.9, 1.7±2.3, and 0.1±0.4, respectively (Figure 2).

**Table 2:** Clinical and radiological findings in patients before the surgery SLR: straight leg raise; n: number; Rt: right; Lt: left

Variables			
Clinical findings			
SLR test (n, [%])			
Positive (Rt)			9 (30)
Positive (Lt)			14 (46.7)
Negative			7 (23.3)
Neurological claudication (n, [%])			
Positive			18 (60)
Negative			12 (40)
Instability pain (n, [%])			
Positive			7 (23.4)
Negative			23 (76.6)
Radiological findings			
Paraspinal muscles area (cm^2^, means±SD)			19.6±4.8
Disc height (mm^2^, means±SD)			8.8±2.8
Grade MODIC change (n, [%])			
0			7 (23.3)
I			5 (16.7)
II			13 (43.3)
III			5 (16.7)
Pfirrmann grading system (n, [%])			
III			4 (13.3)
IV			14 (46.7)
V			12 (40)
Degeneration in adjacent discs (pos n, [%])			28 (93.3)
Osteophyte in vertebral body (pos n, [%])			17 (56.7)
Effusion in vertebral facet (pos n, [%])			9 (30)

Table 3 summarizes clinical outcome trends for patients. After surgery, statistics revealed a significant decrease in ODI scores (P=0.001). Additionally, we used a multivariate linear regression model to determine the factors associated with improved clinical features following surgery. In this regard, we observed a significant correlation between younger age (β: -0.044, P =0.026), low BMI (β: -0.268, P=0.017), absence of neurological claudication (β: -1.035, P=0.043), and improvement in radicular pain. Furthermore, none of the patients' radiologic findings demonstrated a significant correlation with radicular pain improvement.

**Table 3:** Clinical outcomes of patients evaluated in the study VAS: Visual Analogue Score; ODI: Oswestry Disability Index.

Variable	Preoperative data	1 day after surgery	4 weeks after surgery	12 weeks after surgery	24 weeks after surgery
Radicular pain (VAS, means±SD)	8.8±1.15	1.63±0.67	1.1±1	1±0.3	1±0.4
Back pain (VAS, means±SD)	3.47±2.93	3.23±1.67	1.4±1.1	1.03±1	0.9±1
ODI score (means±SD)	29.8±5.2	6.27±2.3	3.73±2.9	1.7±2.3	0.1±0.4
Disability level, n (%)					
No disability	0	7 (23.3)	15 (50)	24 (80)	30 (100)
Mild disability	0	23 (76.7)	15 (50)	6 (20)	0
Moderate disability	4 (13.3)	0	0	0	0
Severe disability	21 (70)	0	0	0	0
Completely disabled	5 (16.7)	0	0	0	0

In non-radiologic variables, higher BMI (β: -0.610, P=0.011) and instability pain (β: 3.226, P=0.006) were associated with improvement in postoperative low back pain. Meanwhile, an increase in the diameter of paraspinal muscles at the level of the damaged disc was associated with a faster postoperative recovery of low back pain (β: 0.45, P=0.021). When the same analysis was performed on the ODI score, it was discovered that higher disc height was associated with a more significant decline in the ODI score following surgery (β: 1.038, P=0.003).

## Discussion

We compared the ODI questionnaire and VAS scores of patients undergoing bilateral discectomy for low back and radicular pain preoperatively and during the 4th, 12th, and 24th week postoperatively against MRI findings in this study. The ODI is widely regarded as the gold standard for low back functional outcome measures [16].

The results indicate that patients who undergo discectomy surgery experience a significant reduction in radicular pain (P =0.001). Furthermore, disability due to low back pain decreased significantly in these patients, following surgery and at the fourth, 12th, and 24th-week follow-up (P =0.001). There was a significant correlation between younger age, lower BMI, the absence of neurological lameness, and a more rapid recovery from radicular pain following surgery. This finding is supported by the fact that being overweight and obesity can impede the process of recovery. As we can see in a study conducted by Rihn et al., obese patients benefited less from lumbar disc herniation surgery and conservative treatment [17]. Additionally, a significant correlation was observed between a lower BMI and postsurgical improvement in back pain. The findings of a study conducted by Rihn et al. supported the role of overweight/obesity in the stability of postsurgical back pain [17].

Due to the aging population in developing countries and physically demanding work, lumbar disc herniation has become the most common reason for spine surgery [18]. Paresthesia is triggered by disc damage caused by the protrusion of a nerve root from the spinal canal. This involvement may result in organ dysfunction, including erectile dysfunction and sphincter disorders. L4-L5 disc involvement results in the lower back and pelvic pain, referred pain (sciatica) in the lower extremities, and numbness and tingling in the anterior thigh area [10]. Additionally, L5-S1 disc involvement will result in excruciating pain in the pelvis and lower limbs, numbness in the lateral thigh and anterior leg [16].

It is discovered that instability pain is associated with greater improvement in back pain in these patients. This concern may be justified in light of the significant impact discectomy surgery has on pain relief in individuals who have suffered from mechanical pain.

Our findings indicated no correlation between MRI findings and low back or radicular pain; however, there was a correlation between ODI and MRI disc height. As a result, patients with a higher disc height had better surgical outcomes. Budiono et al. recently described this finding, indicating that higher disc height is a prognostic factor in patients suffering from L5-S1 degenerative disc disease [19].

Heo et al. evaluated 11 patients with bilateral lumbar disc herniation of the L4-L5 or L5-S1 segments who underwent surgery using a percutaneous biportal endoscopic approach. After one week, the mean VAS scores for the back and both legs had decreased significantly (P< 0.05), as had the mean preoperative Oswestry disability scale score (P< 0.05) [20]. These findings corroborate ours; however, the correlation between these findings and MRI findings was not evaluated.

Aljanabi et al. evaluated the short-term clinical outcomes of open limited discectomy for lumbar disc prolapse in 42 patients using the Japanese Orthopedic Association Score (JOAS) and MacNab criteria, demonstrating that both scores improved significantly (P<0.001), highlighting the efficacy of this method [21]. These findings corroborated our study, which established that this method is beneficial for pain relief. However, this study did not evaluate the MRI results either.

Li et al. described the technical details of unilateral percutaneous transforaminal endoscopic lumbar discectomy (PTELD) and compared unilateral versus bilateral PTELD for L3/4 or L4/5 lumbar disc herniation with bilateral symptoms in a randomized clinical trial [15].

According to the MacNab standard, their findings indicated that both groups experienced a significant postoperative improvement in VAS and ODI scores and similar clinical outcomes. However, the VAS score for back pain one day after surgery was significantly lower in the Unilateral-Approach group than in the Bilateral-Approach group (P<0.05). Although the results agree with ours, this study divided the patients into two groups, which was not done in our study.

Based on our findings, the association between patients' radiologic findings and radicular pain improvement is insignificant, implying that Modic and Pfirrmann changes do not affect patient improvement. Prior research has produced conflicting findings on the subject. While it has been reported that patients with Modic grade I change to experience the same level of pain relief as those without Modic changes, several studies indicate that patients with Modic or Pfirrmann changes may experience more residual pain or have a greater risk of recurrent disc herniation [22-25].

Several studies supporting the latter assertion are retrospective, making consensus difficult and evoking the need for a comprehensive prospective investigation.

We recognize that including only patients who underwent a single surgical technique may constrain the range of findings. Larger sample sizes as well as longer follow-up periods could be used in future studies.

## Conclusions

The use of discectomy in patients with lumbar disc herniation L4-L5 or L5-S1 significantly alleviates postoperative and follow-up low back and radicular pain. Age, BMI, neurological claudication, instability pain, and paraspinal muscle diameter played a role in postoperative pain relief. Increased disc height was associated with a more rapid decline in the ODI score. Subsequent studies with larger sample sizes as well as longer follow-up are recommended to determine the effect of other radiological findings on predicting patient improvement.
